# Perceptions of Parenting in Daily Life: Adolescent-Parent Differences and Associations with Adolescent Affect

**DOI:** 10.1007/s10964-021-01489-x

**Published:** 2021-09-04

**Authors:** Loes H. C. Janssen, Bart Verkuil, Lisanne A. E. M. van Houtum, Mirjam C. M. Wever, Bernet M. Elzinga

**Affiliations:** 1 Department of Clinical Psychology, Leiden University, Leiden, the Netherlands; 2 Leiden Institute for Brain and Cognition (LIBC), Leiden, the Netherlands

**Keywords:** Parenting, Experience sampling method (ESM), Daily life, Discrepancies, Adolescent affect, Perception

## Abstract

Adolescents can perceive parenting quite differently than parents themselves and these discrepancies may relate to adolescent well-being. The current study aimed to explore how adolescents and parents perceive daily parental warmth and criticism and whether these perceptions and discrepancies relate to adolescents’ daily positive and negative affect. The sample consisted of 80 adolescents (*M*_age_ = 15.9; 63.8% girls) and 151 parents (*M*_age_ = 49.4; 52.3% women) who completed four ecological momentary assessments per day for 14 consecutive days. In addition to adolescents’ perception, not parents’ perception by itself, but the extent to which this perception differed or overlapped with adolescents’ perception was related to adolescent affect. These findings highlight the importance of including combined adolescents’ and parents’ perspectives when studying dynamic parenting processes.

## Introduction

Although an important developmental task for adolescents is to become more autonomous and independent, a warm and supportive relationship with parents remains key for adolescent mental health (Steinberg and Silk 2002). What a warm and supportive relationship with parents entails, however, is not so clear-cut, as adolescents and parents can perceive or experience parenting behavior quite differently. For instance, an adolescent might perceive the parent as critical or even rejecting, while the parent may experience his or her own behavior as constructive. Differences between these perspectives of parenting (also referred to as incongruence or discrepancies) have been found to relate to adolescent mental well-being (De Los Reyes et al. 2019; Hou et al. 2019). Most research on discrepancies in parenting in general as well as in relation to adolescent well-being, however, is based on retrospective self-report questionnaires, with recall bias possibly affecting these reports. Moreover, most previous studies focused on cross-sectional or longitudinal designs with macro time intervals (i.e., months or years), while parenting processes evolve dynamically within a family and may change in the daily flow of life (Keijsers and Van Roekel 2018). It remains unclear to what extent adolescents’ and parents’ perspectives of parenting differ on a more micro-level (i.e., on a daily basis) and whether and how these relate to fluctuations in adolescent affect. While changes in mood, such as increases in negative mood and mood instability, can represent normative development for adolescents, it could also be a precursor for psychological problems such as internalizing problems (Maciejewski et al. 2019; Maciejewski et al. 2017). Therefore, the current study aimed to describe how adolescents and their parents (both mothers and fathers) perceive parenting behavior in daily life based on intensive longitudinal data collection, using ecological momentary assessments (EMA; Stone and Shiffman 1994). Additionally, it was explored whether adolescents’ and parents’ perceptions of daily parenting and discrepancies between these perspectives were related to adolescent daily positive and negative affect.

### Adolescent and Parent Perceptions of Parenting Behavior

Recent meta-analyses (Hou et al. 2019; Korelitz and Garber 2016), based on studies using retrospective reports of parenting, have shown that differences between adolescents’ and parents’ perceptions of parenting are quite common. That is, convergence between parent and adolescent reports of several aspects of parenting behavior (i.e., warmth, psychological control) is generally low, with only small correlations between reports of adolescents and parents. Overall, parents view their own parenting behavior as more favorable (more supportive and less negative) than adolescents (De Haan et al. 2018; Hou et al. 2019; Korelitz and Garber 2016). Moreover, parent-adolescent dyads can also vary substantially, with some dyads reporting only few differences while other dyads differ widely in their perceptions (e.g., De Los Reyes et al. 2010; De Los Reyes and Ohannessian 2016; Lippold et al. 2013). To date, it is unclear to what extent the findings on discrepancies based on macro-scale retrospective reports can be generalized to daily life. Furthermore, most existing research has focused on the mother-adolescent dyad, while the family systems theory argues that adolescent-mother and adolescent-father dyads represent distinct but related subsystems (Restifo and Bögels 2009). Research suggests also that mothers and fathers serve different and unique roles in parenting their adolescents (e.g., Lamb and Lewis 2013). Mother-child relationships have been characterized by warmth and support, whereas fathers seem to provide more instrumental care (Youniss and Smollar 1985). Studies indeed showed that mothers are more emotion-directed and supportive than fathers during adolescence (De Goede et al. 2009; Mastrotheodoros et al. 2018). However, parenting studies including fathers are scarce, let alone research on daily parenting. Therefore, the first aim of the current study was to describe adolescents’ and both mothers’ and fathers’ perceptions of parenting in daily life, and potential discrepancies between them. During adolescence, parenting characterized by warm and supportive behavior contributes to the development of a positive self-view, while parenting characterized by criticism and rejection engenders more negative self-views (McCranie and Bass 1984), which might increase vulnerability to depression (Garber and Flynn 2001). This study therefore assessed both positive and negative aspects of parenting with parental warmth referring to showing acceptance, emotional closeness, and positive involvement toward the adolescent (Gladstone and Parker 2005) and parental criticism referring to expressing negativity, dissatisfaction or less responsiveness to an adolescent (Harris and Howard 1984). Gaining insight into these fluctuating processes could contribute to a more comprehensive understanding of parenting and the discrepancies in daily life.

### The Link Between Discrepancies in Parenting and Adolescent Well-Being

Despite the fact that it is increasingly acknowledged that differences between adolescents’ and parents’ perceptions not just represent reporter bias or measurement error (De Los Reyes 2011), but yield valuable information (De Los Reyes and Ohannessian 2016), not many studies yet have investigated to what extent the discrepancies additionally relate to adolescent well-being. These discrepancies might either indicate a normative developmental process related to adolescent autonomy development (De Los Reyes and Ohannessian 2016). In this process, adolescents start to re-evaluate family relationships (Smetana et al. 2006), which may lead to different perceptions in parents and adolescents. However, it may also indicate problems in family functioning processes (De Los Reyes and Ohannessian 2016), such as a misfit between adolescents’ needs and parents’ demands as proposed in the theoretical models on goodness of fit (Eccles et al. 1993; Lerner et al. 1986). In this study, it was therefore tested if, and to what extent, discrepancies are related to adolescents’ well-being, when assessed in daily life.

To date, the interpretation of the findings of the few studies that examined whether and how discrepancies relate to adolescent well-being has been hindered by the usage of different analytic approaches (i.e., difference scores, latent difference scores or interaction terms). A meta-analysis showed that, based on retrospective studies using difference scores, larger discrepancies between parents’ and adolescents’ reports of parenting behavior were related to more adolescent maladjustment (Hou et al. 2019). Specifically, if adolescents perceived parenting more negative (but not more positive) relative to parents, the discrepancy was related to more adolescent negative outcomes (Hou et al. 2019; Rote and Smetana 2016). However, the difference score approach has been criticized for various reasons (see i.e., De Haan et al. 2018). The use of interaction terms in a regression analysis (also known as polynomial regression analysis) has been suggested as an alternative in order to examine not only whether differences between reports relate to outcome variables, but whether these differences relate to the outcome in addition to main effects of individual reports (Laird and De Los Reyes 2013). Results of the retrospective studies that used this approach focused on negative aspects of parenting and indicated for instance that *congruence* of more negative perceptions on parenting or family functioning was related to more adolescent maladjustment (Hou et al. 2019; Van Petegem et al. 2019), but also that high levels of adolescents’ depressive symptoms were related to *incongruence* of father-adolescent reports of negative interactions, with adolescents reporting high and fathers low negative interaction (Nelemans et al. 2016). These results not only suggest that it is important to take into account both congruence and incongruence, but also to examine adolescent-mother and adolescent-father dyads separately. To facilitate the interpretation of the results, it can be insightful to combine polynomial regression with response surface analysis (RSA; Edwards 2002). This approach uses a three-dimensional surface to assess and visualize the association between adolescents’ and parents’ reports of parenting and the outcome variables (see Schönbrodt et al. 2018). Thus, the second aim of this study was to explore whether and how congruence and incongruence between adolescents’ and parents’ reports of daily parenting relate to adolescent daily affect by combining multilevel polynomial regression analyses and RSA. Moreover, in contrast to the previous studies on discrepancies, the current study not only assessed adolescents negative affect, but also positive affect. More insight into the impact of discrepancies between adolescent-parent perceptions of day-to-day parenting on adolescent well-being might ultimately help to inform (preventive) interventions.

## The Current Study

Previous studies on adolescents’ and parents’ perceptions of parenting, discrepancies, and its relation to adolescent well-being focused on cross-sectional or longitudinal designs with macro time intervals and retrospective questionnaires. By using EMA the current study, therefore, aimed to describe to what extent both adolescents and their parents (mothers and fathers) differ or overlap in their perceptions of parental warmth and criticism in daily life (Aim 1). Based on previous meta-analyses, it was expected that adolescents’ and parents’ perceptions of daily parental warmth and criticism would differ substantially, with parents reporting more positive about their own parenting (more warmth and less criticism) than adolescents (Hypothesis 1). The current study furthermore aimed to explore whether congruence and incongruence in adolescents’ and parents’ reports of daily parental warmth and criticism are related to adolescent positive and negative affect in daily life (Aim 2). Based on prior work, it was expected that, on average, *congruent* adolescent-parent reports on high parental criticism and low parental warmth on a given day would relate to more adolescent negative affect and less positive affect on that day (Hypothesis 2a). Moreover, it was expected that, on average, *incongruent* adolescent-parent reports with adolescent reporting more parental criticism and less parental warmth than parents on a given day would relate to more negative affect and less positive affect on that day (Hypothesis 2b). Daily parental warmth and criticism of mothers and fathers was examined separately.

## Methods

### Sample

Data were used from RE-PAIR (Relations and Emotions in Parent Adolescent Interaction Research), a Dutch multi-method two-generation study examining the bidirectional interplay between parent-child interactions and adolescent mental well-being by comparing families with an adolescent with a current major depressive disorder or dysthymia to families with an adolescent without psychopathology. The complete RE-PAIR study consisted of four parts: online questionnaires, a research day at the lab, 2 weeks of EMA, and an Magnetic Resonance Imaging (MRI)-scan session with the adolescent and one parent. The current study used a subsample and only included families with an adolescent without psychopathology and focused on the EMA part of RE-PAIR.

#### Inclusion

Families were included in the study in case the adolescent and at least one of the primary caregivers wanted to participate in the study, and had a good command of the Dutch language. Further inclusion criteria for adolescents were: being aged between 11 and 17 years, living at home with at least on primary caregiver, and going to high school or higher education. Families were excluded if adolescents had a current mental disorder, a history of major depressive disorder or dysthymia, or a history of psychopathology in the last 2 years. Adolescent psychopathology was assessed at the research day during a face-to-face interview using the Semi-Structured Interview of the Kiddie-Schedule for Affective Disorders and Schizophrenia—Present and Lifetime Version (K-SADS-PL; Reichart et al. 2000). For parents, no other in- or exclusion criteria were specified.

Of the 187 families that were interested in participating in RE-PAIR, 87 families were eligible and agreed to participate and a research day was scheduled. Of these families, 4 families (4.6%) canceled the research day and did not participate, 3 adolescents (3.4%) were excluded based on psychopathology (2 adolescents), and still being in primary school (1 adolescent). The final sample of RE-PAIR consisted of 80 families with a total of 233 participants (80 adolescents, 153 parents). Two fathers (1.3%) did not participate in the EMA part of RE-PAIR, resulting in a final sample for the current study of 231 participants (80 adolescents, 151 parents). Sample demographics are presented in Table 1. The majority of adolescents (97.5%) and parents (94.7%) were born in the Netherlands. Adoptive, foster, and stepparents (*n* = 12) were allowed to participate if they were involved in the upbringing of the adolescent for at least 5 years and if adolescents perceived the parent as a primary caregiver. For reasons of clarity, they will be referred to as mothers and fathers from here onwards.

**Table 1:** Sample demographics

Variables	*N*	
Adolescents		
Gender, % Female, (*n*)	80	63.8 (51)
Age (years), *M* (SD)^a^	80	15.9 (1.35)
Highest level of education, % (*n*)	80	
Vocational education		12.5 (10)
Advanced secondary education		33.8 (27)
Pre-university education		45.0 (36)
Secondary vocational education		6.3 (5)
Higher professional education		2.5 (2)
Living situation	80	
With biological mother		6.3 (5)
With biological mother and father		80.0 (64)
Other^b^		13.8 (11)
Parents		
Gender, % Female, (*n*)	151	52.3 (79)
Age (years), *M* (SD)^a^	151	49.0 (5.87)
Highest level of education, % (*n*)	151	
No diploma		0.7 (1)
Lower vocational education		7.3 (11)
Intermediate vocational education		25.8 (39)
Higher vocational education or scientific education (university)		66.2 (100)
Relationship with child–mother, % (n)	79	
Biological parent		96.2 (76)
Stepparent		–
Foster parent		2.5 (2)
Adoptive parent		1.3 (1)
Relationship with child–father, % (n)	72	
Biological parent		87.5 (63)
Stepparent		8.3 (6)
Foster parent		2.8 (2)
Adoptive parent		1.4 (1)

^a^Age at research day

^b^Other options were parent and stepparent, alternating between father and mother, or living with adoptive/foster parents

### Procedure

Families were recruited via networks of employees of Leiden University, flyers at public places, and advertisements in (online) media. Families interested in participating could contact the RE-PAIR research team via the website, telephone, or mail. Information letters were sent to the families and subsequently researchers called parents and adolescents to provide more information and administer screening questions. If all inclusion and no exclusion criteria were met, an appointment was scheduled for a research day in Leiden. All participants signed informed consent. If adolescents were younger than 16 years of age, parents with legal custody also signed informed consent for the adolescent. During the research day, adolescents and parents received face-to-face instructions about the EMA procedure and researchers assisted in installing the Ethica Data application. Each family member also received written instructions and their individual account information. Generally, the EMA started the next Monday after the research day, however in case of holidays and exam weeks of adolescents EMA started the first Monday thereafter.

#### EMA

Participants filled out questionnaires on their own smartphone using the Ethica app for 14 consecutive days between 7AM and 9.30PM on weekdays and 9AM and 9.30PM on weekend days according to a standardized trigger schedule. Participants received four questionnaires each day (56 in total), signaled by a notification, and were instructed to complete the questionnaires as quickly as possible. All questionnaires consisted of questions on their whereabouts, affect, and contact with others. The first questionnaire of each day was sent at 7AM on weekdays and 9AM during weekend days and expired after 120 min. The second and third questionnaires were sent at a random time point, with the second between 12AM and 1PM, and the third between 4PM and 7PM. Both expired after 60 min. The last questionnaire of each day was sent to adolescents at a random time point between 8.15PM and 8.45PM and to parents between 9PM and 9.30PM, both expired after 180 min. The first questionnaire of each day additionally included questions about sleep and the last questionnaire of each day about self-image, parenting, and substance use (e.g., coffee, alcohol) throughout the day. The questionnaires consisted of minimal 14 items, 13 closed and 1 open, and maximal 45 items, 44 closed and 1 open. Number of items depended on role (parent or adolescent), branching, and type of questionnaire (morning, day, or evening). On average, filling out the questionnaires took adolescents 2.21 min per questionnaire (SD = 2.73), and parents 2.66 min per questionnaire (SD = 2.50). Researchers monitored the EMA by checking daily whether participants received and completed questionnaires and were available for questions or problems via WhatsApp, telephone, and mail. On day 4, 7, and 11 of the EMA an update was sent to each participant about the personal adherence (percentage of completed questionnaires) as motivation. On the last day of the EMA, a message was sent to thank participants and remind them of the scheduled phone call after the EMA to evaluate. Participants did not receive automatic reminders for the questionnaires. The EMA of RE-PAIR was conducted in the period between September 2018 and November 2019. As compensation for EMA, parents received €20,- and adolescents €10,-. In addition, four gift vouchers of €75,- were raffled based on compliance.

#### Compliance

In the current study, a total of 4480 questionnaires were planned and 4348 (97.1%) were delivered to the 80 adolescents. Not all questionnaires were sent due to technical errors of the software application or smartphones of the participants. Adolescents fully completed 2954 (67.9%) of the delivered questionnaire (*M* = 36.92 completed, SD = 11.27, Min/Max = 3/55). Adolescent daily affect scores were based on these assessments. Daily parenting was only assessed in the last questionnaire of the day. A total of 1120 questionnaires were planned at the end of each day and 1085 (96.9%) were delivered to the 80 adolescents. Adolescents fully completed 885 (81.6%) questionnaires (*M* = 11.06 completed, SD = 3.10, Min/Max = 1/14). For parents, a total of 2114 questionnaires were planned at the end of each day and 2070 (97.9%) were delivered. Parents fully completed 1881 (90.9%) of the delivered questionnaires (*M* = 12.46 completed, SD = 1.93, Min/Max = 5/14). Several reasons for non-compliance were reported by participants in evaluation phone calls after the EMA part: being at school/work, sleeping late, studying or being on the road. Although some EMA studies use a minimum compliance rate for inclusion, recent evidence suggests that this may lead to inclusion biases. When using compliance thresholds in the analyses potentially valuable data could be omitted (Jacobson 2020). Therefore, no participants were excluded based on missing data and all completed EMA data was retained for analyses.

### Measures

#### Daily parenting

In the last questionnaire of each day, adolescents indicated whether they spoke to a parent during that day and with whom (i.e., mother, father, stepmother, stepfather). In 99.8% of the completed questionnaires, adolescents spoke to one or more parents during that day and these questionnaires were used for the analyses. Adolescents rated parental criticism and parental warmth for each parent they spoke to by answering the questions “Throughout the day, how critical was your mother/father toward you?” and “Throughout the day, how warm/loving was your mother/father toward you?” Answers were given on a seven-point Likert type scale with answer categories ranging from 1 (*not at all)* to 7 (*very*). Only adolescents’ answers about parents who participated in the EMA were included.

Similarly, parents indicated whether they spoke to the participating adolescent in the last questionnaire of each day. In 93.1% of the completed questionnaires, parents spoke to their adolescent and these questionnaires were used for the analyses. Parents rated their own behavior by answering the questions “Throughout the day, how critical were you toward your child?” and “Throughout the day, how warm/loving were you toward your child?” Answers were given on a seven-point Likert type scale with answer categories ranging from 1 (*not at all)* to 7 (*very*). Higher scores indicated more daily parental criticism and parental warmth for parents and adolescents.

#### Daily affect

Adolescents rated their own momentary affect states four times a day with an adapted and shortened four-item version of the Positive and Negative Affect Schedule for Children (PANAS-C; Ebesutani et al. 2012; Watson et al. 1988). Two positive affect states (*happy* and *relaxed)* and two negative affect states (*sad* and *irritated*) were assessed by asking: “How do you feel at this moment?” followed by:”Happy”, “Relaxed”, “Sad”, or “Irritated”. Answers were given on a seven-point Likert type scale with answer categories ranging from 1 (*not at all)* to 7 (*very*). A mean score per affect state per day was calculated. To create a daily positive affect scale, an average score of the two daily positive affect states was calculated, with the two items being strongly correlated with each other at the between person-level, *r*(1051) = 0.667, *p* < 0.001, and moderately at the within-person level*, r*(1051) = 0.428, *p* < 0.001. A mean score of the two daily negative affect states was calculated to create a daily negative affect scale, with the two items also being strongly correlated with each other at the between person-level, *r*(1051) = 0.701, *p* < 0.001, and moderately at the within-person level, *r*(1051) = 0.351, *p* < 0.001. Higher scores represented higher levels of daily positive and negative affect.

### Strategy of Analyses

Descriptive information of study variables was provided on person-mean scores of daily parental warmth and criticism, and adolescent daily positive and negative affect. Between-person and -dyad correlations were calculated based on person-mean scores and within-person and -dyad correlations were calculated based on daily fluctuations around the mean. Normal distribution of variables and equality of variances was checked and when assumptions were not met, appropriate nonparametric tests were used to examine to what extent adolescents’ and parents’ person-mean scores of parenting differed or overlapped (aim 1).

Given the nested structure of the data (repeated measures within persons), multilevel models were specified by using the multilevel package version 2.6 (Bliese 2016) with ML estimation in R Version 3.6.1 (R Core Team 2019). Multilevel models using ML estimation and including all available data should result in unbiased estimates (Little 1995). A total of eight models were built with separate models for mothers and fathers, daily parental warmth and criticism, and daily positive and negative affect. First, two intercept only models were specified to split the total variance in adolescent daily positive and negative affect into stable between-person differences and within-person fluctuations (results in Appendix 1). Second, adolescents’ and parents’ reports of daily parenting were centered on the dyad level, in line with steps proposed by (Nestler et al. 2019). That is, per dyad, the average of the person-mean scores of adolescent and parent reports of parental warmth and criticism was calculated. The centered scores represent the deviation of individual scores from this dyad mean. Centering is important for interpretation of the results since the two predictors then have the same scale midpoint (Edwards 2002). Based on these centered predictor scores, squared terms, and interaction terms between adolescent and parent reports were computed. The centered scores of daily parenting reported by the adolescent and parent were added to the model in the third step.

To examine whether congruence and incongruence in adolescents’ and parents’ reports of daily parental warmth and criticism related to adolescent positive and negative affect in daily life (aim 2), multilevel polynomial regression models were specified by adding the squared and interaction terms in addition to the centered scores of daily parenting of adolescents and parents. The regression coefficients of these models were used for the response surface analyses. In order to illustrate and promote interpretation of the model results, the response surface parameters were used to generate a response surface pattern plot which represents the three-dimensional relation between the two predictor variables (i.e., daily parental warmth reported by adolescents and mothers) and the outcome variable (i.e., adolescent daily negative affect) (Barranti et al. 2017; Nestler et al. 2019) by using the RSA package (version 0.10.4; Schönbrodt and Humberg 2021). For instance, a graphical representation of the three-dimensional relation between fluctuations of adolescents’ and parents’ reports of daily parental warmth and fluctuations in daily negative affect include a line of congruence (i.e., where the values of the two predictor variables perfectly match) and a line of incongruence (i.e., where the values of one predictor are the opposite of the other predictor). The plots represent effects for the average dyads (without taking into account variation between dyads). The four response surface parameters (a1-a4) were calculated based on the unstandardized multilevel polynomial regression coefficients. Specifically, the first two coefficients evaluate statistically whether the slope of the line of congruence (LOC) is linear (a1), which would indicate a linear additive relationship between for instance adolescents’ and parents’ reports of daily parental warmth and daily negative affect, or curvilinear (a2), which would indicate that there is curvilinearity in the relationship between for instance adolescents’ and parents’ reports of daily parental warmth and daily negative affect. The other two coefficients evaluate whether the slope of the line of incongruence (LOIC) is linear (a3), which would indicate that there is a discrepancy effect on the outcome variable in one specific direction, or curvilinear (a4), which would indicate that there is a discrepancy effect on the outcome variable, regardless of the direction. All four parameters were used to examine whether congruence and incongruence between adolescents’ and parents’ reports of daily parenting related to adolescent daily affect. Again, these steps were followed for all eight models.

## Results

### Preliminary analyses

#### Between-person

Table 2 provides descriptive statistics of study variables and between-person (i.e., adolescent reports of affect and parenting) and between-dyad correlations (i.e., adolescent and mother reports of daily parenting of mother) based on person-mean scores. Mothers reported on average significantly more daily parental warmth than fathers, but no significant difference was found between mothers and fathers in daily parental criticism (see Appendix 2 for results and differences between adolescent boys and girls). All between-person correlations between adolescents’ reports of daily parental warmth and criticism of both parents and adolescent daily positive and negative affect were significant (all *p*’s < 0.01) and in the expected direction. For instance, adolescents who reported more daily parental warmth also reported more daily positive affect. As expected, adolescents’ reports of daily parental warmth and daily parental criticism were significantly (negatively) correlated. Interestingly, no significant between-dyad correlations were found between parents’ reports of daily parenting and adolescent daily affect.

**Table 2:** Descriptive statistics and bivariate correlations of study variables

	Descriptives					Between-person and between-dyad correlations									
Variables	*n*	*M*	SD	Min	Max	1	2	3	4	5	6	7	8	9	10
1. Person mean daily positive affect AA	80	5.46	0.77	3.61	6.90										
2. Person mean daily negative affect AA	80	1.52	0.63	1.00	4.21	−0.621***									
3. Person mean daily parental warmth AM	79	5.88	0.81	3.62	7.00	0.553***	−0.375**								
4. Person mean daily parental criticism AM	79	2.06	0.99	1.00	5.00	−0.323**	0.443***	−0.535***							
5. Person mean daily parental warmth AF	72	5.77	0.98	1.38	7.00	0.559***	−0.302**	0.779***	−0.350**						
6. Person mean daily parental criticism AF	72	1.90	0.93	1.00	4.70	−0.317**	0.410***	−0.365**	0.824***	−0.398**					
7. Person mean daily parental warmth MM	79	5.68	0.68	3.67	7.00	0.077	−0.085	0.423***	−0.434***	0.223*	−0.357**				
8. Person mean daily parental criticism MM	79	2.47	0.94	1.07	4.90	−0.121	0.122	−0.226*	0.309**	−0.320**	0.240*	−0.488***			
9. Person mean daily parental warmth FF	72	5.36	0.76	3.50	6.92	0.215	−0.194	0.206	−0.234*	0.223	−0.310**	0.297*	−0.319**		
10. Person mean daily parental criticism FF	72	2.49	0.93	1.00	5.14	−0.213	0.041	−0.063	0.077	−0.232	0.117	−0.127	0.444***	−0.198	

The person means represent the average scores across all assessments per individual

*AA* adolescent about self, *AM* adolescent about mother, *AF* adolescent about father, *MM* mother about own behavior, *FF* father about own behavior

*p < 0.05; **p < 0.01; ***p < 0.001

#### Within-person

To gain more insight into the daily fluctuations in parenting and affect, within-person and within-dyad correlations were calculated (i.e., daily deviations from the person-mean) (see Table 3). Fluctuations in adolescents’ reports of daily parenting of both mothers and fathers were significantly related to fluctuations in adolescent daily positive and negative affect in the expected direction, with the exception of daily parental criticism of fathers. This indicates, for instance, that on days when adolescents reported that their mothers showed more parental warmth, adolescents also reported more positive affect. The strength of the within-person correlations overall was weaker than the between-person correlations (i.e., almost all significant within-person correlations were low, *r* < 0.300). Additionally, intradyad correlation coefficients were calculated to examine the associations between fluctuations of daily parenting at the dyad level (see Appendix 3). The correlation coefficients indicated that dyads differed with regard to both the direction as well as the strength of the intradyad correlation. To further illustrate the daily fluctuations per dyad in parenting reported by adolescent and parent and adolescent affect, plots per dyad were made (see Appendix 4).

**Table 3:** Within-person and within-dyad correlations of study variables

	1	2	3	4	5	6	7	8	9	10
1. Person mean daily positive affect AA										
2. Person mean daily negative affect AA	−0.568***									
3. Person mean parental warmth AM	0.100**	−0.083*								
4. Person mean parental criticism AM	−0.084*	0.081*	−0.218***							
5. Person mean parental warmth AF	0.119**	−0.183***	0.508***	−0.074*						
6. Person mean parental criticism AF	−0.067	0.043	−0.131***	0.512***	−0.259***					
7. Person mean parental warmth MM	0.016	−0.027	0.148***	−0.096**	0.114**	−0.095*				
8. Person mean parental criticism MM	−0.061	0.062	−0.160***	0.235***	−0.114**	0.088*	−0.258***			
9. Person mean parental warmth FF	0.095**	−0.058	0.098*	−0.122**	0.158***	−0.140***	−0.035	−0.067		
10. Person mean parental criticism FF	0.002	−0.041	0.078	0.041	−0.147***	0.171***	−0.083*	0.080*	−0.236***	

The person means represent the average scores across assessments per individual

*AA* adolescent about self, *AM* adolescent about mother, *AF* adolescent about father, *MM* mother about own behavior, *FF* father about own behavior

*p < 0.05; **p < 0.01; ***p < 0.001

### Main Analyses

**Fig. 1:**
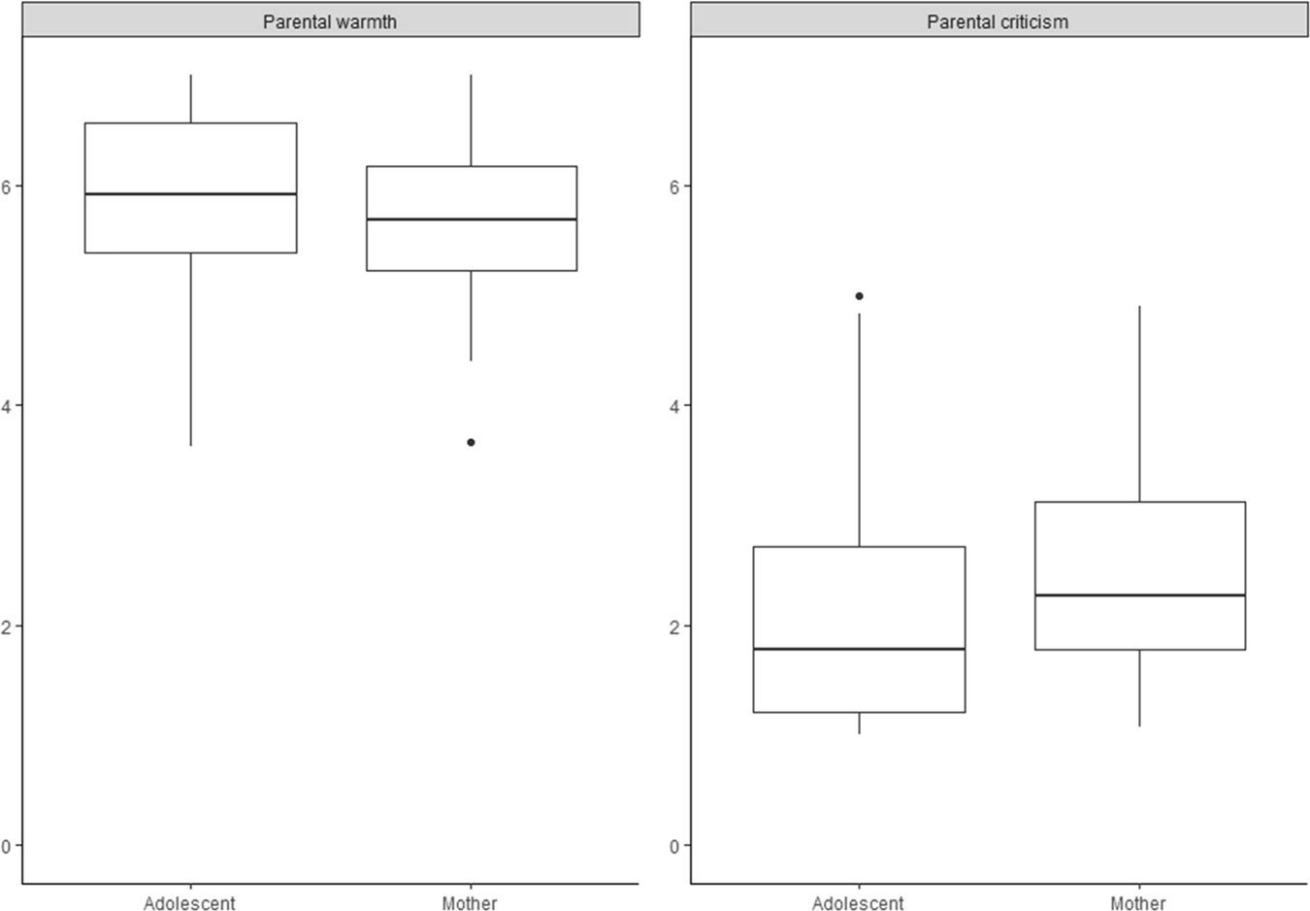
Box plots illustrating the significant differences between adolescents’ and mothers’ person-mean scores of daily parental warmth and criticsm

**Fig. 2:**
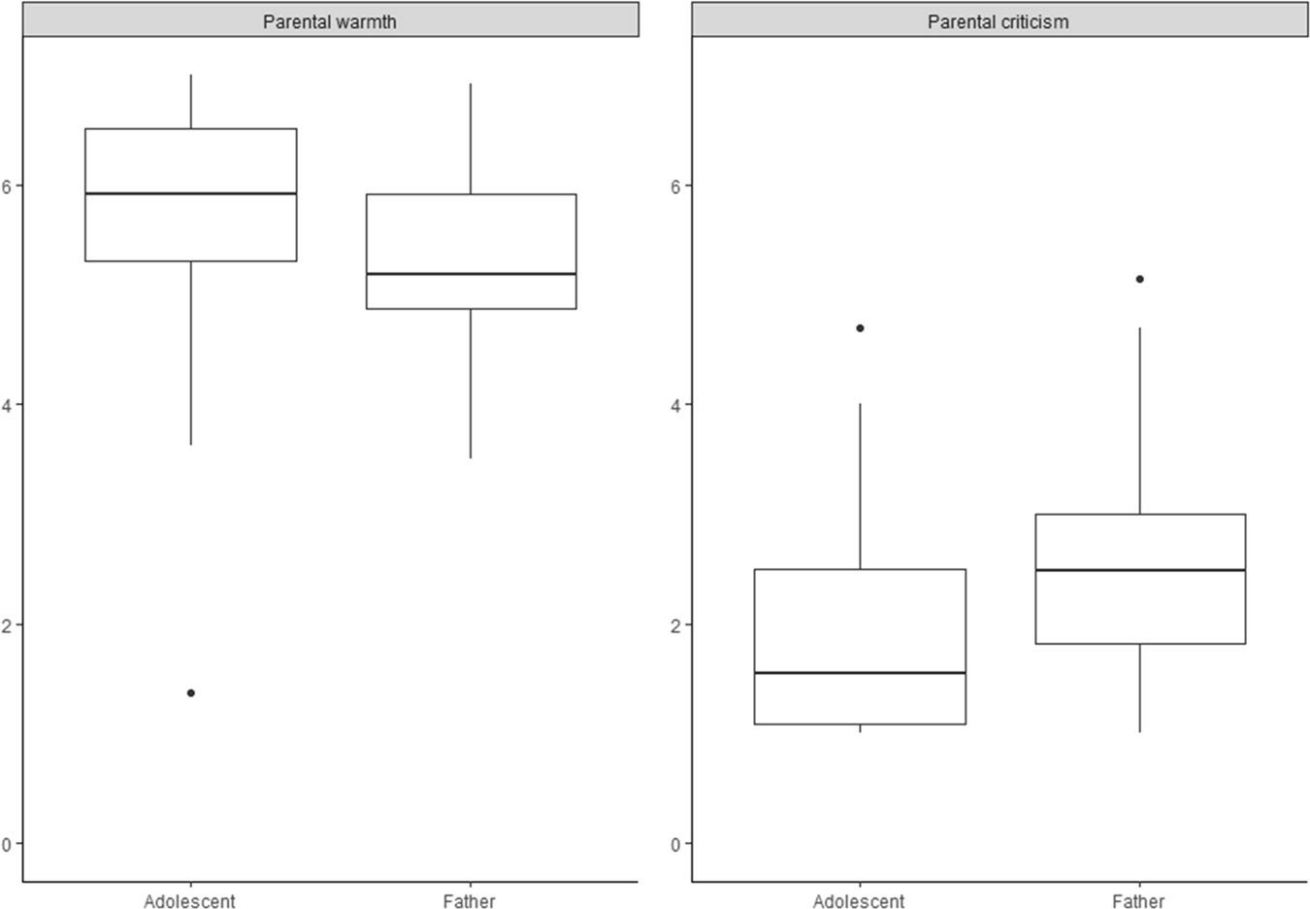
Box plots illustrating the significant differences between adolescents’ and fathers’ person-mean scores of daily parental warmth and criticsm

To examine the first aim, whether adolescents’ and parents’ person mean-level reports of daily parenting differed from each other, paired Wilcoxon’s signed rank tests were used. In line with the expectations, reports of adolescents and parents of daily parental behavior differed significantly, however, not in the expected direction. Adolescents reported significantly higher levels of daily parental warmth than mothers (*z* = −2.300, *p* = 0.021) and fathers (*z* = −3.479, *p* < 0.001), and significantly lower levels of daily parental criticism of both their mothers (*z* = −3.640, *p* < 0.001) and fathers (z = −3.857, *p* < 0.001), see Figs. 1 and 2. Thus, in general, adolescents reported more positively on daily parenting of both their parents than mothers and fathers themselves. To describe the occurrence of these discrepant reports between adolescents and parents of parenting in daily life, adolescents’ and parents’ reports of parenting were compared per day and an aggregated mean difference score per dyad was calculated. These results showed substantial between-dyad variation. In some dyads, adolescents indeed reported more positively than their mothers and fathers on daily parenting, while in other dyads adolescent-parent reports were relatively similar or adolescents reported more negatively on daily parenting than mothers and fathers (see Appendix 5). There was also within-dyad variation representing daily fluctuations. That is, even though a parent-adolescent dyad may have relatively similar scores averaged across two weeks, there are also days on which they differed.

In order to examine the second aim of the study, assessing concurrently whether congruence and/or incongruence between adolescents’ and parents’ reports of daily parental warmth and criticism are related to adolescent daily positive and negative affect, multilevel polynomial regression analyses and RSA were used. Multilevel models including adolescents’ and parents’ reports of daily parenting were first specified (see Appendix 6). Adolescents’ reports of daily parental warmth and criticism were significantly related to adolescent daily positive and negative affect (*p*’s < 0.050), except adolescents’ reports of daily parental criticism of fathers which were not related to daily negative affect. With regard to parents’ reports, only fathers’ reports of daily parental warmth were significantly related to adolescent daily negative affect (*B* = −0.057, *p* = 0.023) and daily positive affect (*B* = 0.078, *p* = 0.020), in addition to adolescents’ reports of daily parental warmth of fathers. That is, adolescents reported on average more negative affect on days when not only adolescents perceived their fathers as showing less parental warmth, but also when fathers themselves reported showing less parental warmth. Mothers’ reports of daily parenting were not related to adolescents’ daily affect, when taking into account adolescents’ reports. Next, the squared and interaction terms between adolescent’ and parent’ reports were added to the models. The unstandardized regression coefficients of these multilevel polynomial regression models were used to calculate the RSA parameters. These parameters in turn were used for the response surface plots to illustrate the results for interpretation. It is important to be cautious when interpreting these plots, since the corners are often extrapolations where no actual observations exist (Tufte 2001).

#### Daily negative affect

The results of the multilevel polynomial regression analyses on daily negative affect and response surface parameters are presented in Table 4.

**Table 4:** Results of multilevel polynomial regression analyses and response surface parameters of adolescent-reported and parent-reported daily parenting related to daily negative affect

	Parental warmth mothers	Parental warmth fathers	Parental criticism mothers	Parental criticism fathers
Multilevel polynomial regression coefficients				
*b*^1^ - adolescent report	−0.067*	−0.079**	0.043*	0.027
*b*^2^ - parent report	0.026	0.000	0.020	−0.034
*b*^3^ - adolescent report^2^	0.033	0.006	−0.005	−0.005
*b*^4^ - adolescent*parent report	0.046	0.020	−0.005	0.007
*b*^5^ - parent report^2^	0.043*	0.052***	0.002	0.004
Response surface parameters				
*a*^1^ - slope along LOC (x = y)	−0.041	−0.078*	0.064**	−0.007
*a*^2^ - curvature along LOC (x = y)	0.122***	0.078*	−0.008	0.007
*a*^3^ - slope along LOIC (x = − y)	−0.092*	−0.079*	0.023	0.061
*a*^4^ - curvature along LOIC (x = − y)	0.029	0.038	0.003	−0.008

Non-standardized coefficients are presented

*p < 0.05; **p < 0.01; ***p < 0.001

##### Daily parental warmth

**Fig. 3:**
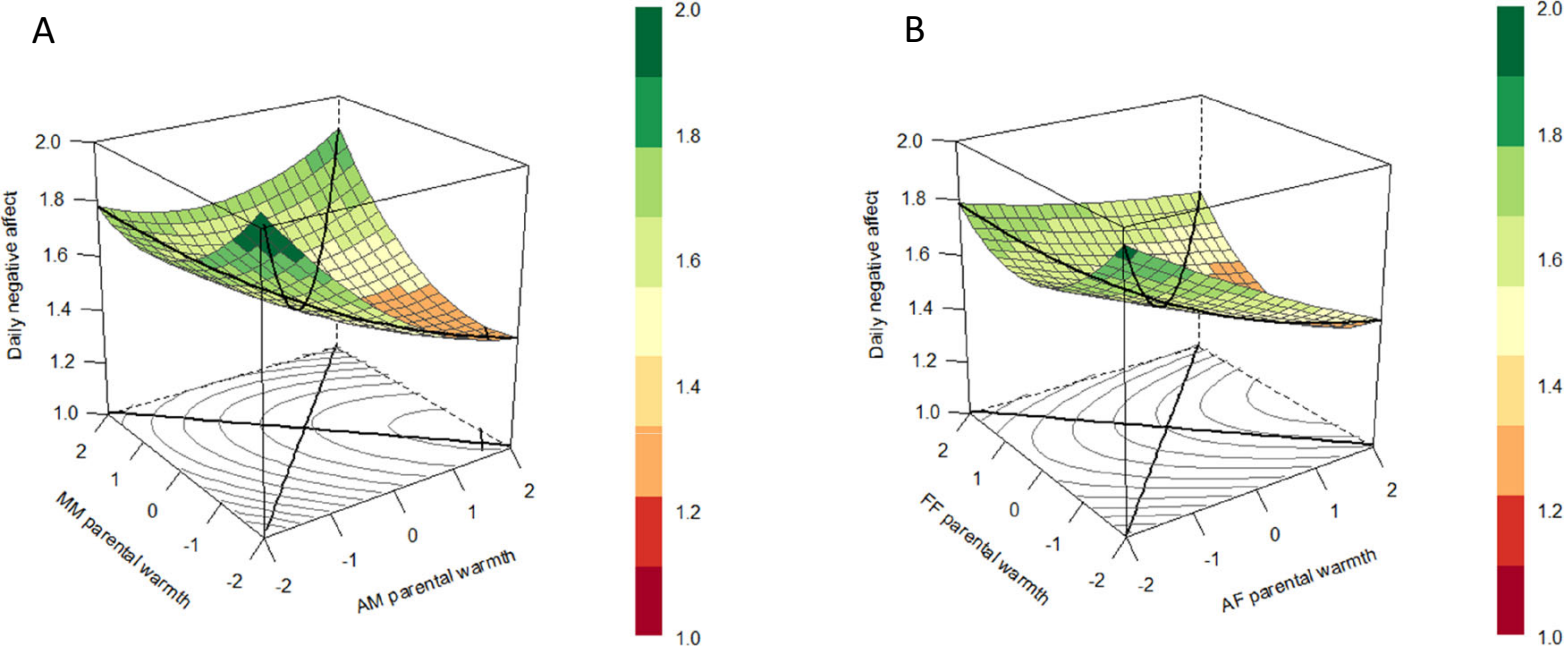
Response surface plots illustrating the association between adolescents’ and mothers’ reports (**A**) and adolescents’ and fathers’ reports (**B**) of daily parental warmth and adolescent daily negative affect with a significant line of congruence, and line of incongruence for the average dyad. *Note*. Centered scores of daily parental warmth of adolescents and parents are presented on the *x*-axis, daily negative affect is presented on the *y*-axis. The colors in the legend represent the amount of daily negative affect which is also shown in the figure

With regard to daily parental warmth of mothers (see Fig. 3A), the curvilinear coefficient related to the LOC was significant, indicating that adolescents reported the least negative affect on days when adolescents’ and mothers’ reports were congruent on average levels (around the dyad mean) of parental warmth. The slope coefficient along the LOIC was also significant, indicating that adolescents reported more negative affect on days when adolescents’ indicated less parental warmth of mothers than mothers themselves. Regarding daily parental warmth of fathers (see Fig. 3B), both the slope and curvilinear coefficient of the LOC were significant, indicating that adolescents reported higher levels of negative affect on days when both fathers and adolescents reported lower levels of parental warmth. This association seems to flatten out at higher levels of parental warmth. In addition, there was also a significant slope coefficient of LOIC. This indicated that adolescents reported more negative affect on days when adolescents’ indicated less parental warmth of fathers than fathers themselves.

##### Daily parental criticism

**Fig. 4:**
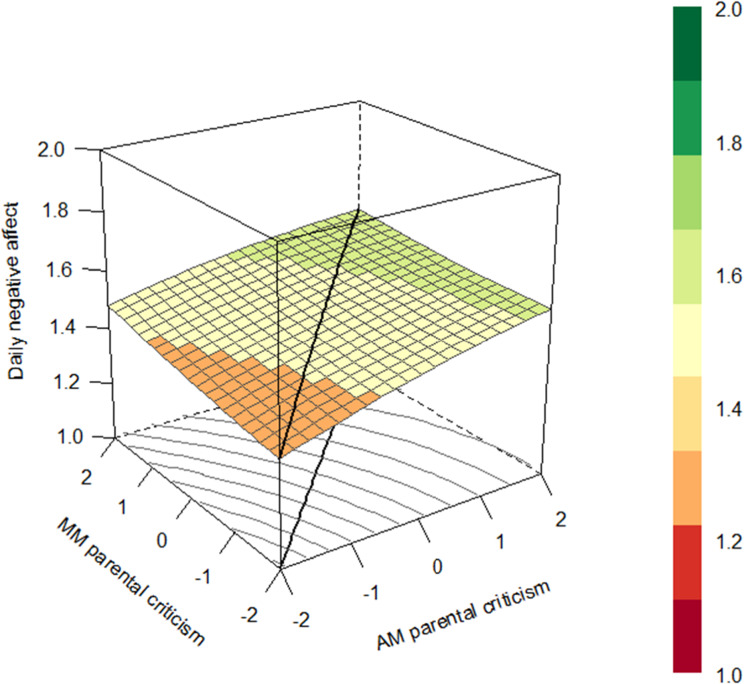
Response surface plot illustrating the association between adolescents’ and mothers’ reports of daily parental criticsm and adolescent daily negative affect with a significant line of congruence for the average dyad. *Note*. Centered scores of daily parental warmth of adolescents and mothers are presented on the *x*-axis, daily negative affect is presented on the *y*-axis. The colors in the legend represent the amount of daily negative affect which is also shown in the figure

With regard to daily criticism of mothers, only the slope coefficient of the LOC was significant (see Fig. 4), indicating that adolescents reported higher levels of negative affect on days when both mothers and adolescents reported higher levels of parental criticism. No significant coefficients were found with regard to daily parental criticism of fathers.

#### Daily positive affect

The results of the multilevel polynomial regression analyses on daily positive affect and response surface parameters are presented in Table 5.

**Table 5:** Results of multilevel polynomial regression analyses and response surface parameters of adolescent-reported and parent-reported daily parenting related to daily positive affect

	Parental warmth mothers	Parental warmth fathers	Parental criticism mothers	Parental criticism fathers
Multilevel polynomial regression coefficients				
*b*^1^ - adolescent report	0.137***	0.061	−0.068**	−0.058*
*b*^2^ - parent report	−0.021	0.030	−0.020	0.018
*b*^3^ - adolescent report^2^	0.017	0.014	0.001	0.018
*b*^4^ - adolescent*parent report	−0.021	−0.038	−0.015	−0.017
*b*^5^ - parent report^2^	−0.001	−0.045*	−0.001	0.007
Response surface parameters				
*a*^1^ - slope along LOC (x = y)	0.116*	0.091	−0.088**	−0.040
*a*^2^ - curvature along LOC (x = y)	−0.005	−0.070	−0.026	0.008
*a*^3^ - slope along LOIC (x = − y)	0.158**	0.031	−0.048	−0.075
*a*^4^ - curvature along LOIC (x = − y)	0.036	0.007	0.014	0.043

Non-standardized coefficients are presented

*p < 0.05; **p < 0.01; ***p < 0.001

##### Daily parental warmth

**Fig. 5:**
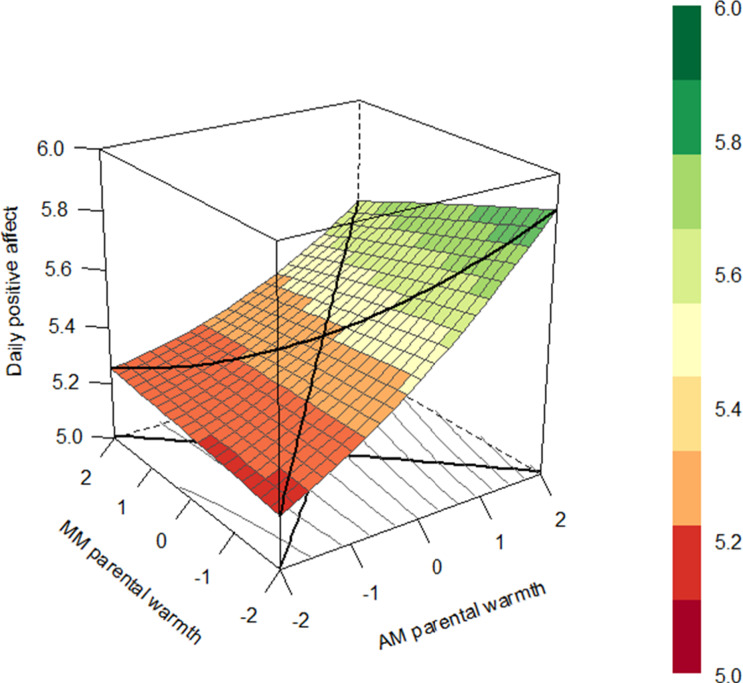
Response surface plot illustrating the association between adolescents’ and mothers’ reports of daily parental warmth and adolescent daily positive affect with a significant line of congruence and line of incongruence for the average dyad. *Note*. Centered scores of daily parental warmth of adolescents and mothers are presented on the *x*-axis, daily positive affect is presented on the *y*-axis. The colors in the legend represent the amount of daily positive affect which is also shown in the figure

The slope coefficients of the LOC and LOIC were both significant regarding daily parental warmth of mothers (see Fig. 5). This indicates that adolescents reported more positive affect on days when both mothers and adolescents reported higher levels of parental warmth. Moreover, adolescents reported more positive affect on days when adolescents’ indicated more parental warmth of mothers than mothers themselves. No significant coefficients were found with regard to daily parental warmth of fathers.

##### Daily parental criticism

**Fig. 6:**
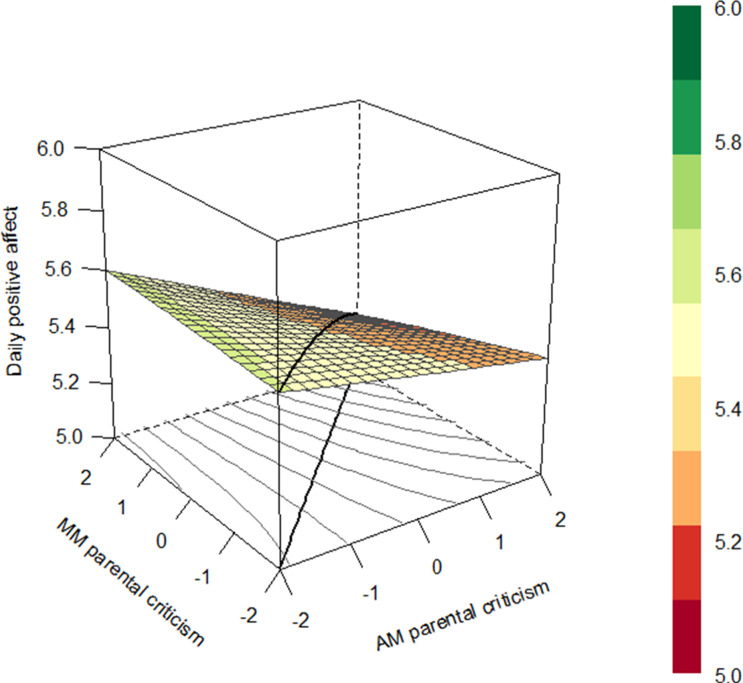
Response surface plot illustrating the association between adolescents’ and mothers’ reports of daily parental criticsm and adolescent daily positive affect with a significant line of congruence for the average dyad. *Note*. Centered scores of daily parental criticism of adolescents and mothers are presented on the *x*-axis, daily positive affect is presented on the *y*-axis. The colors in the legend represent the amount of daily positive affect which is also shown in the figure

The slope coefficient of the LOC was significant concerning daily parental criticism of mothers (see Fig. 6), indicating that adolescents reported lower levels of positive affect on days when both mothers and adolescents reported higher levels of parental criticism. Again, no significant coefficients were found with regard to daily parental criticism of fathers.

## Discussion

Even though an important developmental task for adolescents is to become more autonomous and independent, a warm and supportive relationship with their parents remains essential for their well-being (Steinberg and Silk 2002). Adolescents and parents can perceive their relationship and behavior quite differently, with for instance adolescents perceiving their parents to be less critical than parents see themselves. These discrepancies have been found to relate to adolescent well-being (De Los Reyes et al. 2019; Hou et al. 2019), but previous studies focused solely on classical retrospective reports, while parenting is a dynamic concept that can change in the daily flow of life within a family (Keijsers and Van Roekel 2018). In addition, the majority of studies so far focused on negative aspects of parenting and parenting of mothers. The current study therefore aimed to describe adolescents’ and both mothers’ and fathers’ perceptions of parental warmth and parental criticism in daily life. Additionally, it was examined whether these daily perceptions, congruence, and incongruence between reports were related to adolescent daily positive and negative affect.

Overall, the results showed that not parents’ perspective of daily parenting by itself, but differences and overlap with adolescents’ perspective in addition to adolescent individual reports were of importance for adolescent daily well-being. This was not only the case for negative aspects of parenting but also regarding parental warmth. Considering, for instance, mothers’ perspective and the discrepancy with adolescents’ perspective of daily parental warmth helped to understand why some adolescents showed more daily negative affect and less positive affect. Using more sophisticated methodology such as multilevel polynomial regression analyses and RSA, as suggested by previous studies (Edwards 2002; Schönbrodt et al. 2018), contributed to a more comprehensive understanding of risk factors for more negative and less positive affect in daily life.

### Perceptions of Parenting in Daily Life

Previous studies have shown that generally parents report more positive on their own parenting behavior than adolescents (de Haan et al. 2018; Hou et al. 2019), but these studies focused on retrospective self-reports. The current study aimed to explore the extent to which adolescents and their parents differ or overlap in their perceptions of parental warmth and criticism when zooming in on the daily level. In contrast to the previous findings, results showed that adolescents reported more positively on daily parental warmth and criticism of their mothers and fathers than parents themselves. It should also be noted, however, that there was substantial variation between dyads. In some adolescent-parent dyads (34% adolescent-mother dyads; 50% adolescent-father dyads) adolescents did report more daily parental warmth than their parents, while other adolescent-parent dyads adolescents (20% adolescent-mother dyads; 17% adolescent-father dyads) reported less daily parental warmth criticism compared to their parents. Previous studies already indicated that dyads differ in the specific patterns of divergence (e.g., De Los Reyes et al. 2010; De Los Reyes and Ohannessian 2016; Lippold et al. 2013) and the current findings support this and more importantly show that this is also the case when zooming in to a micro-level (i.e., days).

### Associations of Congruence and Incongruence in Daily Parenting Related to Adolescent Affect

While it is increasingly acknowledged that differences between adolescents’ and parents’ perceptions of parenting yield valuable information (De Los Reyes and Ohannessian 2016), not many studies have yet investigated whether and how the differences and overlap between these perceptions relate to adolescent well-being. The current results indicated that, in line with previous studies, adolescents’ perceptions of parenting were more strongly related to adolescent well-being than parents’ perceptions (Hendriks et al. 2018). Overall, parents’ perceptions of daily parenting were only related to adolescent daily affect when combined with adolescents’ perceptions, but not by itself. With regard to parental criticism, it was found that if adolescents and mothers (but not adolescents and fathers) *agreed* on elevated levels of daily parental criticism this was associated with more daily negative affect and less daily positive affect in adolescents which is in line with a previous study (Nelemans et al. 2016). *Disagreement* between adolescent-mother and adolescent-father reports of daily parental criticism, however, was not related to adolescent daily affect in the current study. This is in contrast to the expectations since discrepancies between adolescent and father reports of negative interactions were related to more adolescent depressive symptoms (Nelemans et al. 2016). A possible explanation for these contradicting findings may be that the previous study retrospectively measured negative interactions in general at a certain time point while the current study included a more fine-grained aspect of negative parenting in daily life. Parental criticism was assessed on multiple consecutive days and therefore takes into account the dynamic process of parenting and adolescents’ affect in daily life (Keijsers and Van Roekel 2018).

The current study additionally examined whether congruence and incongruence between reports of a positive aspect of daily parenting, parental warmth, were also related to adolescent daily affect. As expected, on days when adolescents and mothers (but not adolescents and fathers) *agreed* on lower levels of daily parental warmth adolescents reported lower levels of adolescent positive affect. In contrast to the hypotheses, adolescents reported the least negative affect on days when adolescents and mothers agreed on average levels of parental warmth. This finding might seem somewhat counterintuitive, however, since the current study included daily assessments the results concern daily fluctuations in parenting and affect. Congruent scores at average levels of parental warmth may refer to a certain consistency or stability in parental warmth of mothers. Since inconsistent parenting may impact adolescent well-being negatively (De Los Reyes and Ohannessian 2016), the findings of the current study that consistency (i.e., adolescent-mother agreement on average levels around the dyad mean of parental warmth) related to less adolescent negative affect seems plausible. Moreover, the current study included healthy adolescents and their parents who reported rather high levels of parental warmth. It might be that these average levels of parental warmth are good enough and that more parental warmth may be perceived and experienced as smothering. The results regarding *congruent* adolescent-father reports on daily parental warmth are largely in line with adolescent-mother dyads, but here the curve flattens at higher levels of parental warmth. That is, agreement of adolescents and fathers on lower levels of parental warmth is more strongly related to adolescent negative affect than agreement on higher levels of parental warmth.

With regard to *incongruence* between reports of parental warmth, adolescents reported more daily negative affect on days when fathers and mothers reported more parental warmth than adolescents did. Moreover, adolescents reported more daily positive affect on days when mothers reported less parental warmth than adolescents reported themselves. These results are in line with findings of previous studies using both difference scores (Laird and De Los Reyes 2013) and interaction terms (Nelemans et al. 2016), and support the theoretical models on goodness of fit (Eccles et al. 1993; Lerner et al. 1986). That is, when adolescents’ reports of parental warmth of fathers and mothers are lower than parents’ reports it may indicate that the parental behavior does not fit the needs of an adolescent and this seems to result in more negative affect. Alternatively, a negative mood of adolescents may also have influenced the perception of parenting.

Overall, differences and overlap between adolescents’ and mothers’ perceptions of parenting were more related to adolescent affect in daily life than adolescents’ and fathers’ perceptions. Even though adolescents and fathers in the current study reported to speak to each other a on daily basis, it might be that adolescents spend more time with their mothers than fathers and are thus more affected by mothers (Larson et al. 1996). Moreover, it has been suggested that the quality of relationship between adolescents and mothers and fathers might be different with mothers providing more emotional support and fathers giving more instrumental care (Youniss and Smollar 1985). Interestingly, incongruence and congruence between adolescents’ and fathers’ reports of daily parental warmth were only related to adolescent negative, and not to daily positive affect. Although most studies on adolescent-parent discrepancies focused on negative outcomes or solely included mother-adolescent dyads, the current findings are in line with a prior study, which showed that father-child discrepancies only related to adolescent maladjustment (Hou et al. 2017). Mother-child discrepancies of parenting did relate to positive psychological measures in adolescents, which supports findings of the current study. Despite the additional data needed to strengthen this interpretation, these findings suggest that discrepancies with mothers are of more relevance for adolescent positive well-being than with fathers.

The current study demonstrated the importance of taking into account differences and overlap between adolescents’ and parents’ perceptions in of parenting (both positive and negative aspects of parenting) in addition to individual reports for understanding daily fluctuations in adolescent well-being. This may also provide some first useful insights for preventive interventions. More understanding of how both parents and adolescents perceive certain parental behavior may help them to become more aware of the fact that these perceptions may differ. This could result in a realization for parents that their often well-intended behavior may not suit the needs of an adolescent, but also enable adolescents to better understand their parents’ behaviors and intentions. Becoming more attuned to each other might affect adolescent well-being in a positive manner.

### Strengths, Limitations, and Future Research

The current study used ecologically valid measures of parenting and adolescent affect that minimized recall bias and provided insights into the daily dynamic family life processes. The use of EMA and including both perceptions of adolescents and parents further enabled a more fine-grained exploration of parent-adolescent differences and overlap in perceptions of parenting. This provided some first insights into the substantial between-dyad and within-dyad variation regarding the discrepancies. Moreover, in addition to negative aspects of parenting and adolescent well-being, positive aspects such as parental warmth and adolescent positive affect were also taken into account. The current results supported the importance of including a wider range of parenting behaviors. By using sophisticated analyses, the current study was able to examine whether congruence and incongruence between adolescent-parent reports of daily parenting related to adolescent daily affect in addition to main effects of individual reports. This provided a more detailed representation of daily life of families. Furthermore, fathers were included in the study which enabled assessing these processes in both adolescent-mother and adolescent-father dyads.

The study also has some limitations that generate ideas for future approaches. While the relatively high levels of adolescent daily positive affect and low levels of adolescent negative affect are in line with previous studies (Beyens et al. 2020; Janssen et al. 2021), the sample consisted of a fairly homogeneous sample of healthy adolescents in the Netherlands with highly educated parents. It is therefore unknown to what extent the current findings generalize to more diverse or clinical samples. This should be addressed in future studies. Additionally, it should be acknowledged that the current sample of 80 families was relatively small. Nevertheless, based on a rule of thumb that 550 observations should be sufficient for detecting small effect sizes in RSA (Barranti et al. 2017), the sample is not underpowered with at least 600 observations. Moreover, performing a multilevel model with a sample size of at least 50 units at level 2 should result in unbiased estimates (Maas and Hox 2005) which should to apply to multilevel RSA as well (Nestler et al. 2019). This seems to imply that the minimum of 72 units at level 2 in the current study would suffice, but future research in larger samples is needed to strengthen the findings. Moreover, the discrepancies between adolescents’ and parents’ reports of daily parenting might represent differences between psychometric properties of adolescent versus parent reports (De Los Reyes et al. 2016) and measurement invariance between these reports was not tested in this study. Parents and adolescents, however, answered the exact same questions regarding parenting in the family context, so the discrepant reports are not due to different item content, response options, or context. In addition, the response surface analyses represent effects for the average dyad without taking into account the between-dyad variation, while the current study showed that discrepancies between adolescents’ and parents’ reports varied between-dyads and even within-dyads. However, it was beyond the scope of this study to test this heterogeneity. Future studies should include this as it might provide insights into which adolescents might be affected most by congruence and incongruence between adolescent-parent reports on daily parenting. Another suggestion for future research would be to include person-mean levels of daily parenting as well as the fluctuations in order to gain more understanding of the importance of the stability of parenting for adolescent well-being. Although the current study assessed both adolescent-mother and adolescent-father dyads, since these should be seen as distinct but related subsystems according to the family system theory (Restifo and Bögels 2009), the interrelatedness of these dyads within one family was not taken into account due to the already complex models. Future studies should aim to include adolescents-mother and adolescent-father dyads in a family model to obtain a better understanding of the unique processes within each family. This would also enable testing explicitly for differences between adolescent-mother and adolescent-father dyads. Moreover, it would be interesting to also take into account the actual time spend together by adolescents and their mothers and fathers in order to examine whether this influences the impact of discrepancies on adolescent well-being. A final recommendation for future studies is to assess whether adolescents and parents are aware of the fact that their perceptions of parenting behavior differ, and whether this awareness can be related to adolescent well-being. This knowledge may provide valuable insights that ultimately could inform prevention strategies or interventions in clinical practice.

## Conclusion

It is increasingly acknowledged that differences between adolescents’ and parents’ perceptions of parenting yield valuable information, but few studies have actually examined to what extent the discrepancies relate to adolescent well-being. Moreover, whether earlier findings using retrospective questionnaire data generalize to dynamic daily life processes remains unclear. By using EMA, multilevel polynomial regression analyses and RSA, the current multi-informant study showed that in addition to adolescents’ perspective, not parents’ perspective of own parenting in daily life by itself, but the extent to which this perspective corresponded to or differed from adolescents’ perspective was of importance for adolescent well-being. Both congruence and incongruence between adolescents’ and parents’ reports of daily parental warmth were related to adolescent daily affect. Variation was found between adolescent-mother and adolescent-father dyads, with differences and overlap between adolescents and fathers being only related to adolescent daily negative affect. Incongruence and congruence between adolescents and mothers was related to both daily positive and negative affect. If adolescents and mothers agreed on higher levels of daily parental criticism, adolescents reported more negative and less positive affect. The current study furthermore showed that adolescents’ and parents’ reports of daily parenting differed substantially and varied between- and within-dyads. Taken together, the findings highlight the importance of taking into account the overlap and differences between adolescents’ and parents’ reports of parenting in daily life in relation to adolescent daily affect. Not only to gain more insight into the micro-social processes and fluctuations in adolescent daily affect, but also to ultimately use this valuable information in preventive interventions for families to make parents and adolescents become more attuned to each other.

## Supplementary information

Supplementary Materials
