# Supplementary material for: Perceptions of Parenting in Daily Life: Adolescent-Parent Differences and Associations with Adolescent Affect
Source: J Youth Adolesc. 2021 Sep 4;50(12):2427–43. doi: 10.1007/s10964-021-01489-x (PMC8580902; doi:10.1007/s10964-021-01489-x)
Supplement: Supplementary file 1 — Supplementary Materials [file 10964_2021_1489_MOESM1_ESM.docx]

**Supplementary Materials**

**Perceptions of Parenting in Daily Life:**

**Adolescent-Parent Differences and Associations with Adolescent Affect**

*Journal of Youth and Adolescence*

Loes H. C. Janssen^1,2^, Bart Verkuil^1,2^, Lisanne A.E.M. van Houtum^1,2^, Mirjam C.M. Wever^1,2^, & Bernet M. Elzinga^1,2^

*^1^Department of Clinical Psychology, Leiden University, Leiden, the Netherlands;*

*^2^Leiden Institute for Brain and Cognition (LIBC), Leiden, the Netherlands*

Contact corresponding author [l.h.c.janssen@fsw.leidenuniv.nl](mailto:l.h.c.janssen@fsw.leidenuniv.nl)

**Appendix 1.**

Results of intercept only models of adolescent daily positive and negative affect.

The intercept only model of daily negative affect, without predictors, showed an intraclass correlation (ICC) of 0.566 suggesting that 56.6% of the variance in adolescent daily negative affect was due to differences between adolescents, and the remainder 43.4% due to within-person fluctuations over time. The intercept only model of daily positive affect showed and ICC of 0.585 suggesting that 58.5% of the variance in adolescent daily positive affect was due to differences between adolescents, and the remainder 41.5% due to within-person fluctuations over time.

**Appendix 2.**

Information on differences between mothes and fathers on daily parenting and adolescent boys and girls on daily parenting behavior and daily affect.

Differences between mothers and fathers on age and person-mean levels of daily parenting were assessed. A Welch’s *t*-test showed that fathers were significantly older than mothers (*t* = 3.621, *p*  < .001; fathers *M* = 50.81, *SD* = 6.60; mothers: *M* = 47.43, *SD* = 4.61). An independent sample *t*-test showed that mothers reported significantly more daily parental warmth than fathers (*t* = -2.765, *df* = 149, *p*  = .006; mothers: *M* = 5.68, *SD* = 0.68; fathers *M* = 5.36, *SD* = 0.76). A Mann-Whitney U test showed that there was no significant difference between mothers and fathers regarding daily parental criticism (*z* = -0.123, *p*  = .902).

Differences between adolescent boys and girls on age, person-mean levels of adolescent daily affect, and person-mean levels of daily parenting were assessed. An independent sample *t*-test showed that boys reported significantly higher levels of daily positive affect than girls (*t* = 2.427, *df* = 78, *p*  = .018; boys *M* = 5.73, *SD* = 0.81; girls: *M* = 5.30, *SD* = 0.71). Mann Whitney U tests showed that there were no significant differences between boys and girls regarding age (*z* = -1.746, *p*  = .080), daily negative affect (*z* =-1.832, *p*  = .067), and daily parental warmth and daily parental criticism of mothers and fathers (all *p*’s ≥ .050).

**Appendix 3.**

Information on intradyad correlation coefficients

One correlation coefficient per dyad was calculated, representing the extent to which adolescents’ reports of daily parental warmth of mother are related to mothers’ reports of own daily parental warmth in the same family (intradyad correlation). Descriptive statistics of these dyad specific correlations are presented in the Table below and indicated that adolescents and parents seem to agree most on daily parental criticism of mothers. Differences between dyads are found, as indicated by the range of the observed intradyad correlations, with regard to both the direction as well as the strength of the correlation. For instance, reports of daily parental warmth of mothers by adolescents and mothers are positively related in some families, while in others these are related negatively.

Descriptive statistics of intradyad correlations

|  | *N* | *M* | *SD* | *Min* | *Max* |
| --- | --- | --- | --- | --- | --- |
| Daily parental warmth mother | 64 | 0.15 | 0.33 | -0.64 | 0.85 |
| Daily parental criticism mother | 66 | 0.24 | 0.36 | -0.71 | 1 |
| Daily parental warmth father | 59 | 0.14 | 0.42 | -1 | 1 |
| Daily parental criticism father | 53 | 0.11 | 0.48 | -1 | 0.93 |

*Note.* We were unable to calculate within-dyad correlations for all families due to a lack of variance in adolescents’ or parents’ reports of parenting, or due to many missing values.

**Appendix 4.**

**
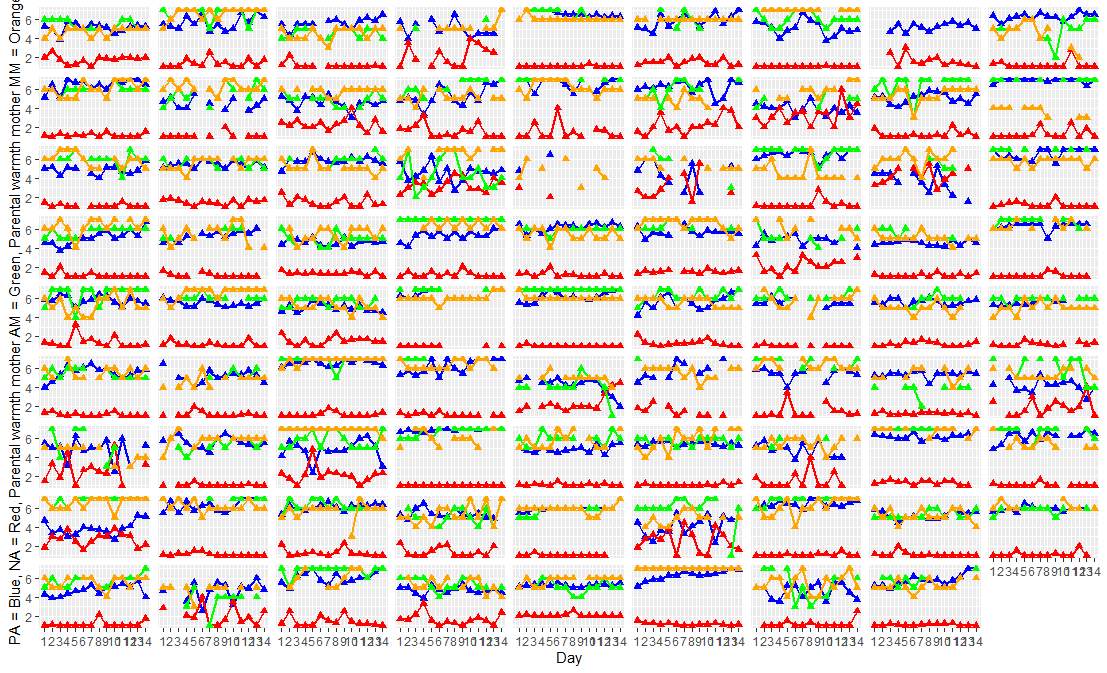
**

Illustrations per dyad of fluctuations in daily parental warmth of mothers and adolescent daily negative affect. Each grid represents a dyad.

PA = positive affect, NA = negative affect, AF = adolescent about father, MM = mother about own behavior


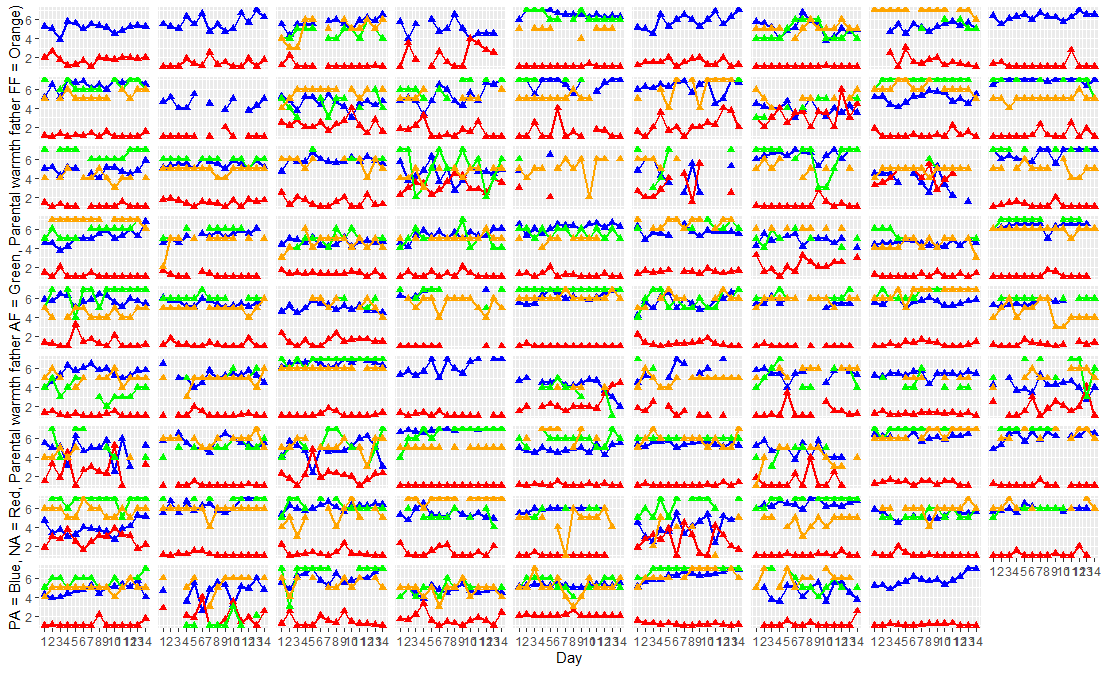


Illustrations per dyad of fluctuations in daily parental warmth of fathers and adolescent daily affect. Each grid represents a dyad.

PA = positive affect, NA = negative affect, AF = adolescent about father, FF = father about own behavior


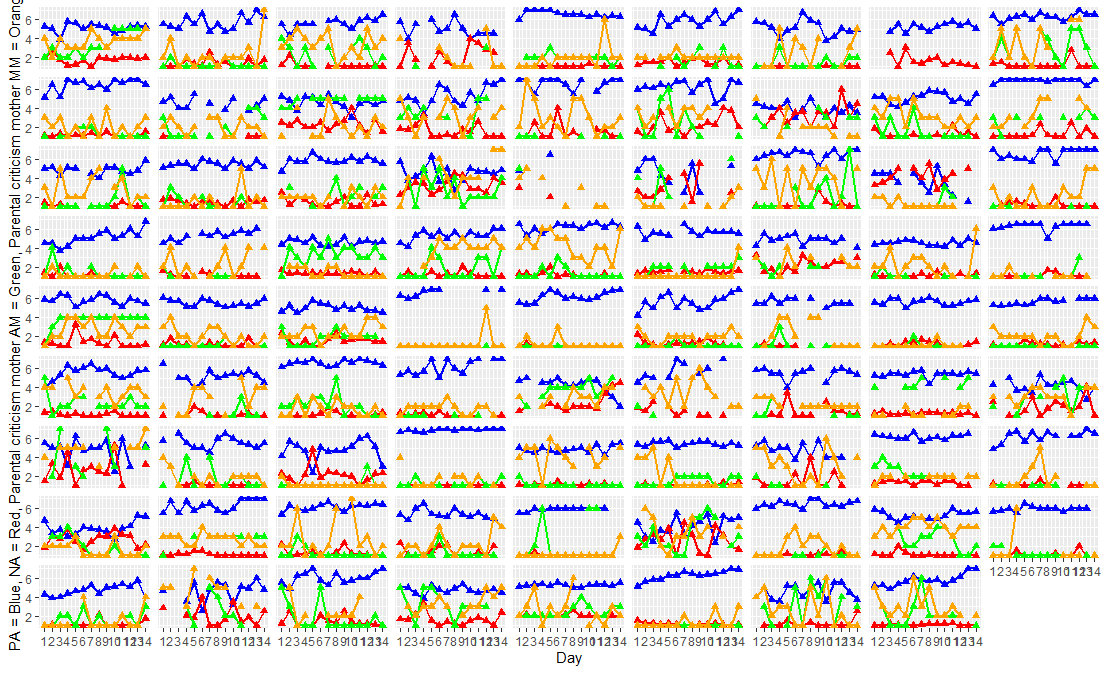

Illustrations per dyad of fluctuations in daily parental criticism of mothers and adolescent daily negative affect. Each grid represents a dyad.

PA = positive affect, NA = negative affect, AF = adolescent about father, MM = mother about own behavior


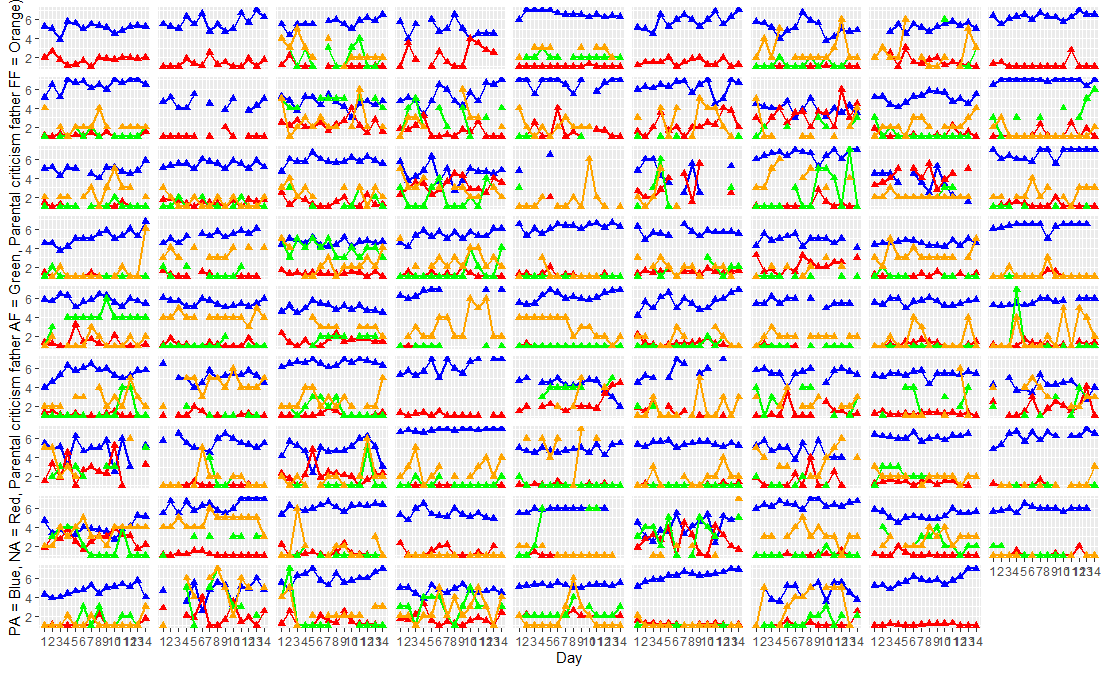


Illustrations per dyad of fluctuations in daily parental criticism of fathers and adolescent daily affect. Each grid represents a dyad.

PA = positive affect, NA = negative affect, AF = adolescent about father, FF = father about own behavior

**Appendix 5.**

Information on between-dyad variation in occurrence of discrepancies


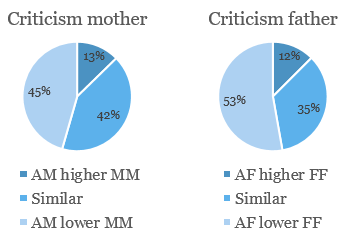

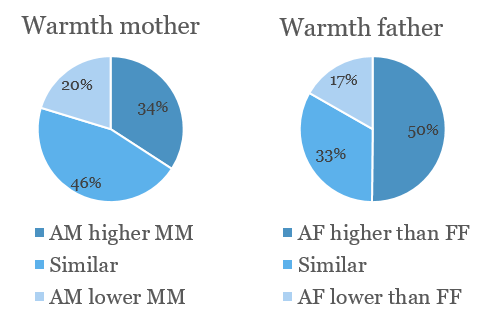
Adolescents’ and parents’ reports of parenting were compared per day. An aggregated mean difference score per dyad was calculated to examine how often adolescent reports were higher than, equal to, and lower than parent reports. A difference more than half a standard deviation between adolescent and parent reports was used as a cut-off (Shanock et al.2010).

Specifically, 34.2% and 50.0% of the adolescents reported higher levels of daily parental warmth of mothers and fathers respectively, 45.6% and 33.3% of the adolescents reported relatively similar scores on daily parental warmth as compared to their mothers and fathers, whereas 20.3% and 16.7% of the adolescents reported lower levels of daily parental warmth than their mothers and fathers. Regarding parental criticism, 12.7% and 12.5% of the adolescents reported higher levels of daily parental criticism of mothers and fathers respectively, 41.8% and 34.7% of the adolescents reported relatively similar scores on daily parental criticism as compared to their mothers and fathers, whereas 45.6% and 52.8% of the adolescents reported lower levels of daily parental warmth than their mothers and father.

**References**

Shanock, L. R., Baran, B. E., Gentry, W. A., Pattison, S. C., & Heggestad, E. D. (2010). Polynomial regression with response surface analysis: a powerful approach for examining moderation and overcoming limitations of difference scores. *Journal of Business and Psychology, 25*, 543–554. doi:10.1007/s10869-010-9183-4

**Appendix 6.**

Results of models on the association between adolescent reports of daily parenting and daily affect

Results of models on the association between adolescent and parent reports of daily parenting and daily negative affect

|  | Parental warmth mothers | | | | Parental warmth fathers | | | | Parental criticism of mothers | | | | | | Parental criticism of fathers | | | |  |
| --- | --- | --- | --- | --- | --- | --- | --- | --- | --- | --- | --- | --- | --- | --- | --- | --- | --- | --- | --- |
|  | Estimate | *SE* | *p* |  | | Estimate | *SE* | *p* | |  | Estimate | *SE* | *p* |  | | Estimate | *SE* | *p* | |
| Intercept | 1.519 | 0.069 | < .001 |  | | 1.507 | 0.075 | < .001 | |  | 1.510 | 0.070 | < .001 |  | | 1.519 | 0.074 | < .001 | |
|  |  |  |  |  | |  |  |  | |  |  |  |  |  | |  |  |  | |
| Adolescent report | -0.090 | 0.024 | < .001 |  | | -0.087 | 0.023 | < .001 | |  | 0.039 | 0.018 | .035 |  | | 0.027 | 0.018 | .146 | |
| Parent report | 0.002 | 0.024 | .927 |  | | -0.057 | 0.025 | .023 | |  | 0.027 | 0.015 | .064 |  | | -0.028 | 0.016 | .086 | |
| Between dyad variance | 0.349 |  |  |  | | 0.376 |  |  | |  | 0.352 |  |  |  | | 0.366 |  |  | |
| Within dyad variance | 0.202 |  |  |  | | 0.162 |  |  | |  | 0.203 |  |  |  | | 0.167 |  |  | |
|  |  |  |  |  | |  |  |  | |  |  |  |  |  | |  |  |  | |
| N dyads | 79 |  |  |  | | 72 |  |  | |  | 79 |  |  |  | | 72 |  |  | |
| N observations | 775 |  |  |  | | 617 |  |  | |  | 775 |  |  |  | | 617 |  |  | |

Results of models on the association between adolescent and parent reports of daily parenting and daily positive affect

|  | Parental warmth mothers | | | | Parental warmth fathers | | | | | Parental criticism of mothers | | | | | Parental criticism of fathers | | | |  |
| --- | --- | --- | --- | --- | --- | --- | --- | --- | --- | --- | --- | --- | --- | --- | --- | --- | --- | --- | --- |
|  | Estimate | *SE* | *p* |  | | Estimate | *SE* | *p* |  | | Estimate | *SE* | *p* |  | | Estimate | *SE* | *p* | |
| Intercept | 5.439 | 0.084 | < .001 |  | | 5.456 | 0.094 | < .001 |  | | 5.444 | 0.086 | < .001 |  | | 5.425 | 0.094 | < .001 | |
|  |  |  |  |  | |  |  |  |  | |  |  |  |  | |  |  |  | |
| Adolescent report | 0.131 | 0.030 | < .001 |  | | 0.071 | 0.030 | .017 |  | | -0.072 | 0.023 | .002 |  | | -0.049 | 0.024 | .045 | |
| Parent report | -0.023 | 0.030 | .448 |  | | 0.076 | 0.033 | .022 |  | | -0.017 | 0.018 | .365 |  | | 0.036 | 0.021 | .093 | |
| Between dyad variance | 0.510 |  |  |  | | 0.566 |  |  |  | | 0.535 |  |  |  | | 0.558 |  |  | |
| Within dyad variance | 0.320 |  |  |  | | 0.307 |  |  |  | | 0.323 |  |  |  | | 0.313 |  |  | |
|  |  |  |  |  | |  |  |  |  | |  |  |  |  | |  |  |  | |
| N dyads | 79 |  |  |  | | 72 |  |  |  | | 79 |  |  |  | | 72 |  |  | |
| N observations | 775 |  |  |  | | 617 |  |  |  | | 775 |  |  |  | | 617 |  |  | |
